# Conservative treatment of idiopathic spontaneous pneumoperitoneum in a bedridden patient: a case report

**DOI:** 10.1186/s40792-015-0073-x

**Published:** 2015-08-28

**Authors:** Ryo Tanaka, Hitoshi Kameyama, Masayuki Nagahashi, Tatsuo Kanda, Hiroshi Ichikawa, Takaaki Hanyu, Takashi Ishikawa, Takashi Kobayashi, Jun Sakata, Shin-ichi Kosugi, Toshifumi Wakai

**Affiliations:** Division of Digestive Surgery, Sanjo General Hospital, 5-1-62 Tsukanome, Sanjo, Niigata 955-0055 Japan; Division of Digestive and General Surgery, Niigata University Graduate School of Medical and Dental Sciences, 1-757 Asahimachi-dori, Chuo-ku, Niigata 951-8510 Japan

**Keywords:** Idiopathic spontaneous pneumoperitoneum, Non-surgical pneumoperitoneum, Conservative treatment

## Abstract

Idiopathic spontaneous pneumoperitoneum is a rare condition that is characterized by intraperitoneal gas for which no clear etiology has been identified. We report here a case of idiopathic spontaneous pneumoperitoneum, which was successfully managed by conservative treatment. A 77-year-old woman who was bedridden with speech disability as a sequela of brain hemorrhage presented at our hospital with a 1-day history of abdominal distention. On physical examination, she had stable vital signs and slight epigastric tenderness on deep palpation without any other signs of peritonitis. A chest radiograph and computed tomography showed that a large amount of free gas extended into the upper abdominal cavity. Esophagogastroduodenoscopy revealed no perforation of the upper gastrointestinal tract. The patient was diagnosed with idiopathic spontaneous pneumoperitoneum, and conservative treatment was selected. The abdominal distension rapidly disappeared, and the patient resumed oral intake on the 5th hospital day without deterioration of symptoms. Knowledge of this rare disease and accurate diagnosis with findings of clinical imaging might contribute towards refraining from unnecessary laparotomy.

## Background

Radiographic manifestation of free gas in the peritoneal cavity often suggests intra-abdominal emergencies, including gastrointestinal tract perforation, in which timely surgical intervention is required. Idiopathic spontaneous pneumoperitoneum (ISP) is a rare condition that is characterized by intraperitoneal gas without gastrointestinal tract perforation, for which no clear etiology has been identified [1].

Most cases of spontaneous pneumoperitoneum can be conservatively managed [2]. However, patients with ISP often undergo surgery because diagnosis of ISP preoperatively is difficult, and diagnosis is confirmed by negative findings in laparotomy [3].

We present a case of ISP that appeared in a bedridden patient with cerebral hemorrhage sequela. In this case, conservative treatment was selected and the patient could avoid unnecessary surgery. We also discuss clinical issues in the management of this rare disease.

## Case presentation

A 77-year-old-woman was transferred to our hospital with the chief complaint of abdominal distention, which occurred the day before admission. The patient was bedridden with speech disability as a sequela of brain hemorrhage. She was cared for in a nursing facility and had no other comorbidities on treatment or a history of laparotomy. The patient did not have a fever, decreased appetite, dyspepsia, abdominal pain, vomiting, or constipation in weeks before admission.

**Fig. 1:**
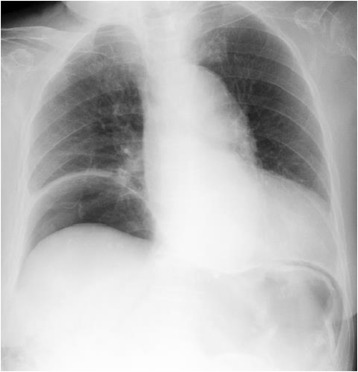
Chest radiograph in the sitting posture. Chest radiograph shows free gas in the right and left subdiaphragmatic regions

**Fig. 2:**
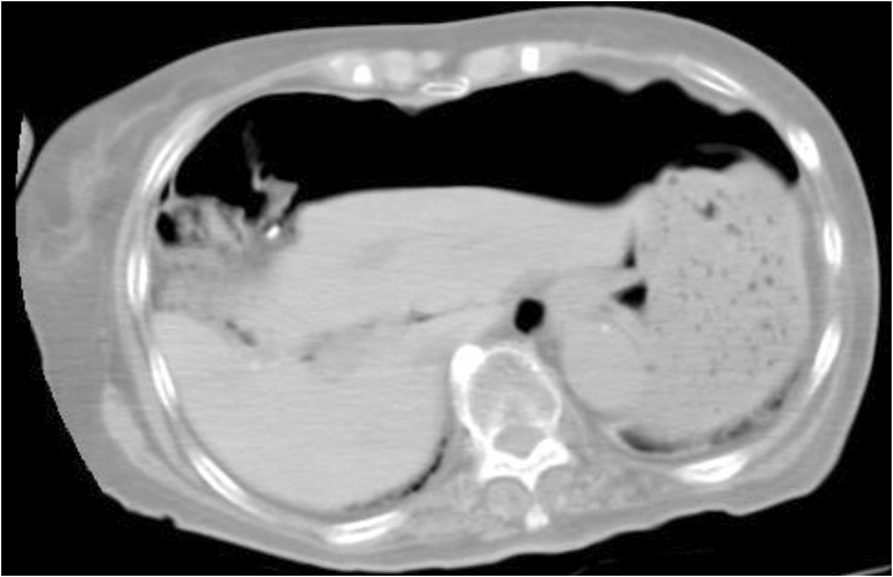
Abdominal computed tomography. Computed tomography findings show a massive pneumoperitoneum localized to the upper abdomen without collection of intra-abdominal fluid, pneumoretroperitoneum, or subcutaneous emphysema

Physical examination showed slight epigastric tenderness on deep palpation, but no peritoneal signs were found. No trauma or subcutaneous emphysema was evident. The vital signs of the patient were as follows: body temperature was 36.9 °C, pulse rate was 90 beats/min, respiration was 18 breaths/min, and blood pressure was 170/108 mmHg. A chest radiograph in the sitting posture showed free gas below the diaphragm (Fig. 1). The patient was suspected as suffering from perforation of the upper gastrointestinal tract, and urgently underwent a computed tomography (CT) scan. The abdominal CT scans revealed large quantities of gas without fluid collection, which were spread in the upper abdomen, mainly at the surface of the liver, but not in the bursa omentalis, lower abdomen, or pelvis (Fig. 2). There were no findings of gastrointestinal wall thickening, distention, edema, or obstruction. In the lower abdomen and pelvis, there were no findings suggestive of lower gastrointestinal tract perforation or gynecological disease. There was no mediastinal air or no findings suggestive of thoracic diseases in the chest CT. Emergent esophagogastroduodenoscopy was undertaken, and this procedure failed to identify any evidence of perforation in the upper gastrointestinal tract. The results of laboratory tests were as follows: the white blood cell count was 8120/mm^3^, hemoglobin was 14.6 g/dl, the serum C-reactive protein level was 0.05 mg/dl, PaCO_2_ was 39.6 mmHg, PaO_2_ was 80.8 mmHg, and base excess was 0.8 mmol/l.

Based on these findings, we diagnosed the patient with ISP. After obtaining informed consent, we started conservative treatment with parenteral nutrition. The patient showed a markedly prompt recovery. The epigastric tenderness improved within the following day, and abdominal distension disappeared within 2 days. The laboratory data on the 3rd hospital day were normal. Oral intake was resumed on the 5th hospital day. The clinical course was subsequently uneventful, and the patient was transferred to the nursing facility on the 9th hospital day. After 10 months, the patient is doing well, without any signs of recurrence.

### Discussion

Pneumoperitoneum is a result of a gastrointestinal tract perforation in more than 90 % of patients. Pneumoperitoneum without gastrointestinal perforation, also called spontaneous pneumoperitoneum or non-surgical pneumoperitoneum, is an uncommon entity related to intrathoracic, abdominal, gynecological, iatrogenic, and other miscellaneous causes [1, 2]. ISP is an even rarer condition than spontaneous pneumoperitoneum, from which gastrointestinal tract perforation and other known causes of free intraperitoneal gas have been excluded [1].

To characterize this rare disease, we searched the PubMed database for papers published in English between Jan. 1, 1990 and Dec. 31, 2014, with the search terms “spontaneous pneumoperitoneum” or “idiopathic pneumoperitoneum”. Only 13 patients with ISP were identified after exclusion of cases of children or neonates. Previously reported clinical features of ISP patients are shown in Table 1 [3–14]. Of the 14 patients including our patient, four were associated with connective tissue disorders. With regard to clinical manifestations, four patients had sudden onset of abdominal pain or distension, and nine were associated with digestive symptoms. Only one patient showed clinical evidence of peritonitis. In all the patients, plain chest or abdominal radiographic examination was performed, which revealed free subdiaphragmatic air. CT examination was performed in only six patients, and four patients of the six were treated conservatively. Six patients underwent emergency surgery to aid diagnosis and to evacuate the intraperitoneal free gas. One patient had a past history of the same experience, and the other three had recurrence of pneumoperitoneum during treatment.

**Table 1:** Clinical characteristics of 14 patients of idiopathic spontaneous pneumoperitoneum

	Year	Age	Sex	Past history	Fever	WBC	Sudden onset	Digestive symptom	Signs of peritonitis	Diagnostic tools			Laparotomy	Recurrence
										Contrast study	Endoscopy	CT		
London et al. [4]	1990	64	F	Scleroderma	−	WNL	−	+	−	+	−	−	−	−
Wang et al. [5]	1993	50	F	Systemic sclerosis	−	WNL	−	+	−	+	−	−	−	+
Tani et al. [6]	1995	70	M	−	+	10200/mm^3^	+	+	+	+	+	+	−	+
Hussain et al. [7]	1995	93	F	Arthritis	−	WNL	−	+	−	−	−	−	−	−
Clements et al. [8]	1996	68	F	Taking NSAIDs	−	WNL	−	−	−	+	+	−	−	−
Mularski et al. [9]	1999	54	M	Bipolar disorder	−	12500/mm^3^	−	−	−	−	−	+	−	−
Mularski et al. [9]	1999	17	F	Raynaud’s phenomenon	−	WNL	−	−	−	−	−	−	+	−
Eslick et al. [3]	2006	75	M	Duodenal ulcer Depression	−	WNL	−	−	−	−	−	+	+	−
Masood et al. [10]	2009	58	F	−	−	WNL	+	+	−	−	−	−	+	−
Mann et al. [11]	2010	88	M	Multiple strokes	−	WNL	−	+	−	−	−	+	−	+
Pitiakoudis et al. [12]	2011	69	F	−	+	15000/mm^3^	+	+	−	−	−	−	+	−
Freitas Jr et al. [13]	2011	63	F	Tuberculosis	−	WNL	+	+	−	−	−	−	+	−
McLaren [14]	2013	46	M	−	−	WNL	−	+	−	−	−	+	+	+
Our case	2014	77	F	Brain hemorrhage	−	WNL	−	−	−	−	+	+	−	−

Fever was defined as a body temperature higher than 37.5 °C. Digestive symptoms include appetite loss, nausea, vomiting, diarrhea, and constipation. Signs of peritonitis were defined as rigidity, muscular guarding, and/or rebound tenderness. Endoscopy means esophagogastroduodenoscopy, and CT means computed tomography

*WBC* white blood cell count, *WNL* within normal limits, *NSAIDs* non-steroidal anti-inflammatory drugs

A detailed history and physical examination can be helpful, and moreover, CT is an indispensable method for the diagnosis of ISP. CT findings predict the site of gastrointestinal tract perforation with 86 % accuracy [15]. If free gas is present only around the liver and stomach and not in the pelvis, proximal gastrointestinal perforation is likely [15, 16]. In the present case, although we did not have confirmation with colonoscopy or exploratory laparotomy, we found that our patient did not have small-intestinal or colorectal perforation based on CT findings. The subsequent course proved this diagnosis. An accurate diagnosis with CT findings should allow surgeons to avoid unnecessary surgery.

Some reports have suggested that patients with asymptomatic spontaneous pneumoperitoneum should be conservatively managed [1, 8, 9]. Moreover, Tani et al. suggested that ISP is amenable to conservative treatment, even when signs and symptoms of peritonitis are present [6]. Taking into account a consensus of conservative treatment for perforated peptic ulcer [17, 18], patients with pneumoperitoneum localized to the upper abdomen, who have mild symptoms and stable hemodynamics, should be initially considered for conservative treatment with anti-ulcer drug, such as H2 blocker or proton pump inhibitor, under frequent and careful clinical monitoring. In doubtful situations, laparoscopic exploration may be a good alternative to laparotomy [19].

## Conclusions

In summary, we have described a case of ISP, which was successfully treated with conservative treatment. Knowledge of this rare disease and accurate diagnosis with findings of clinical imaging might contribute towards refraining from unnecessary laparotomy.

## Consent

Written informed consent was obtained from the patient and the family for the publication of this case report and any accompanying images. A copy of the written consent is available for review by the editor in chief of this journal.

## Abbreviations

CT: computed tomography
ISP: idiopathic spontaneous pneumoperitoneum
